# Outcomes of Coronary Artery Bypass Grafting (CABG) Patients With and Without a History of Liver Transplant

**DOI:** 10.7759/cureus.75820

**Published:** 2024-12-16

**Authors:** Andrej M Sodoma, James R Pellegrini, Samuel Greenberg, Andrej Sodoma, Rezwan Munshi, Richard G Pellegrini, Jaspreet Singh

**Affiliations:** 1 Internal Medicine, South Shore University Hospital, Bay Shore, USA; 2 Internal Medicine, Nassau University Medical Center, East Meadow, USA; 3 Anesthesia and Critical Care, Renaissance School of Medicine at Stony Brook University, Stony Brook, USA; 4 Chemistry Faculty, College of Natural Sciences, Grand Canyon University, Phoenix, USA; 5 School of Medicine, St. George's University, St. George's, GRD; 6 Gastroenterology, Northwell Health, Bay Shore, USA

**Keywords:** acute-on-chronic liver failure, cirrhosis, high-risk cabg, mortality, solid organ transplant

## Abstract

Background: Liver transplant (LT) patients face various challenges, including an increased risk of coronary artery disease (CAD) for a variety of reasons, with 70% of LT recipients having one cardiovascular event. Coronary artery bypass grafting (CABG) remains one of the most commonly performed major surgical procedures in the United States, with 20-30% of LT patients requiring a CABG. Many studies have analyzed when to perform a CABG and CAD workup pre-LT, but this population remains a problem. The patient population is challenging to study due to their rarity and complexity. Our study aimed to compile many patients through the National Inpatient Sample (NIS) database to gauge the outcomes of CABG in patients with and without a history of LT.

Methods: Patients who underwent CABG with or without a history of LT were selected from the NIS from 2008 to 2020. The International Classification of Diseases (ICD) 9 and 10 codes were used to identify suitable records. Primary outcomes of interest were all-cause hospital mortality, shock, acute myocardial infarction, acute kidney injury (AKI), and a composite of these. Secondary outcomes included length of stay and total charges.

Results: A weighted total of 2,407,349 CABG hospitalizations were included in this study. Of these, 1,833 had a history of LT. Overall, patients with a history of LT were more likely to be younger (65.16 vs. 66.16; p<0.001), male (81.6% vs. 73.66%; p<0.001), and more complex (Charlson Comorbidity Index (CCI) 5.89 vs. 4.16; p<0.001) than patients without a history of LT. Patients with a history of LT also had higher rates of diabetes mellitus type 2 (57.02% vs. 43.39%; p<0.001), end-stage renal disease (11.21% vs. 2.95%; p<0.001), and gastroesophageal reflux disease (GERD) (28.39% vs. 21.26%; p<0.001). CABG patients with a history of LT were less likely, however, to have hyperlipidemia (56.72% vs. 74.26%; p<0.001), hypertension (25.95% vs. 58.45%; p<0.001), obesity (19% vs. 23.42%; p=0.046), a history of smoking (12.06% vs. 18.66%; p<0.01), or alcohol use disorder (9.04% vs. 13.44%; p=0.017). We found that patients admitted for CABG with a history of LT had significantly higher adjusted odds of mortality (OR 1.84; p<0.01), AKI (OR 2.65; p<0.001), and composite outcome (OR 2.04; p<0.001). They also experienced a longer length of stay (1.7 days; p=0.02) and greater hospital charges ($26,761; p=0.029).

Conclusion: We found that CABG patients with a history of LT had nearly twofold higher odds of mortality, nearly threefold higher odds of AKI, and twofold higher odds of composite outcomes than CABG patients without LT. This corresponded to longer lengths of stay and increased hospital charges. Patients should require lower thresholds for left heart catheterization and more strict CAD testing before an LT due to the increased risk of adverse outcomes with the current standard of care.

## Introduction

Coronary artery bypass grafting (CABG) is a high-risk surgery for patients used to treat coronary artery disease (CAD). CABG is the standard of care for patients with left main CAD due to overall better outcomes [1]. It involves breaking and opening the sternum; the heart is stopped. When stopping the heart, the patient is connected to a heart-lung bypass machine. Then, a vascular conduit is taken from the patient, and various vessels can be used. The vessel is then transplanted above and below the blocked artery [2]. Patients with end-stage liver disease (ESLD) tend to have CAD for a variety of reasons. Patients with ESLD have high resting myocardial blood flows, which do not respond well to vasodilators. They also have coagulopathy, thrombocytopenia, renal failure, low systemic vascular resistance (SVR), ascites, and hepatorenal syndrome, which can all damage the heart [3,4]. 

Since CAD is a common result in ESLD, before going for a liver transplant (LT), patients must undergo CAD screening. It involves history, exams, electrocardiogram (ECG), transthoracic echocardiogram (TTE), functional testing, and sometimes direct assessment of myocardial perfusion by cardiac imaging [2]. The American Heart Association (AHA)/American College of Cardiology (ACC) guidelines state that the presence of >3 cardiovascular risk factors (>60 years old, diabetes mellitus, smoking, hypertension, dyslipidemia, left ventricular hypertrophy (LVH)) prompts screening by dobutamine stress echocardiography (DSE) or single-photon emission computed tomography (SPECT). However, the exact methods/risk factor thresholds for cardiac imaging have yet to be agreed upon [5,6]. 

This is a problem because cardiovascular events are the leading cause of peri-transplant mortality. LT, in many ways, creates a lot of stress on the heart [7]. When the diseased liver is removed, and the donor's liver is placed, there are reduced venous return, an increase in SVR, a decrease in cardiac output (CO), and an increase in pulmonary capillary wedge pressure (PCWP), described as hemorrhage and reperfusion syndrome, for up to two weeks post-transplant [7-10]. It has been found that cardiac surgery (CS) in severe liver disease before LT has a risk of decompensated liver failure due to cardiopulmonary bypass. However, if planned to be done post-LT, roughly 70% of LT recipients have one cardiovascular event, with a mortality rate between 3% and 11%. Major cardiovascular events are 12-15% higher in patients with CAD post-LT. Most events occur in the first month post-LT [11-14]. The exact treatment plans of these pre-LT and post-LT patients requiring cardiac intervention are not agreed upon. This is partly due to these few patients, which are complicated and rare, requiring multidisciplinary decision-making. Also, there is no agreement on CAD workup pre-transplant or post-transplant. This study uses National Inpatient Sample (NIS) data to gauge the outcomes of CABG in patients with and without a history of LT. It is a retrospective study of many patients from the NIS over a long period, from 2008 to 2020. Previous studies have been unable to concentrate this number of patients with this medical history due to this small patient population's inherent rarity and complexity. This large sample size elucidates the relationship between outcomes of patients receiving CABG with a history of LT.

This article was previously presented as a meeting abstract at Digestive Disease Week (DDW) on May 21, 2024. 

## Materials and methods

Data source

Our retrospective cohort study uses the 2008-2020 NIS, a database provided by the Healthcare Cost and Utilization Project (HCUP) [15]. The NIS is one of the largest databases available in the United States and is maintained by the Agency for Healthcare Research and Quality (AHRQ). Data is gathered from each state that is based on a representation of the patient population and hospital characteristics. The NIS consists of a weighted one-fifth sample of all inpatient hospitalizations in the United States. The data within the NIS includes basic demographic information, clinical details, resource utilization, access to healthcare, charges, patient outcomes, and all International Classification of Diseases (ICD) diagnoses and procedure codes. Data are weighted per the algorithm supplied by the NIS to provide a representative estimate of all relevant hospitalizations in the United States during the study period. Due to its nature as an inpatient database, the NIS does not allow for tracking long-term outcomes after discharge. Like other databases, it may also be vulnerable to errors and omissions in billing codes and demographic data. An institutional review board (IRB) was not required for this study, as the NIS includes patient information that has been de-identified and made publicly available.

Study population and endpoints

Our study included all inpatients who underwent CABG of any number of coronary arteries during this hospitalization (ICD-10 procedure codes: 0210-, 0211-, 0212-, 0213-; ICD-9 procedure codes: 36.1-). Patient records with incomplete demographic information were excluded, which consisted of 61,825 records (300,441 patients) from the no LT group and 39 records (188 patients) from the LT group. Patients with a history of LT before this hospitalization were similarly identified with ICD codes (ICD-10 diagnosis code: Z94.4; ICD-9 diagnosis code: V42.7). Patients undergoing other operations during this operation were not excluded. Our primary endpoint of interest was all-cause hospital mortality. Additional endpoints included common complications of LT, such as acute renal failure, shock, acute myocardial infarction, and a composite of mortality and these morbidities. 

Statistical analysis

The Pearson chi-squared and Student's t-test assessed categorical and continuous variables. After adjusting for baseline characteristics and comorbidities to account for confounding variables, a two-step multivariate regression model was used, emphasizing variables with p<0.05. The first and second steps included variables of age, gender, race, hospital bed size, Charlson Comorbidity Index (CCI), type of insurance, median income based on zip code, day of admission, and hospital region. SAS 9.4 by SAS Institute (Cary, North Carolina, United States) was utilized for all statistical analyses.

## Results

A weighted total of 2,407,349 CABG hospitalizations were included in this study. Of these, 1,833 had a history of LT (0.076%). Patients with a history of LT were more likely to be younger (65.16 years old vs. 66.16 years old; p<0.0001). Patients with a history of LT were more likely to be male (81.6% history of LT were male vs. 73.66% no LT were male; p<0.0004). Patients without a history of LT were more likely to be female (18.39% vs. 26.33%; p<0.0004). LT patients were more likely to be White (83.02% vs. 79.44%; p<0.0882) or Hispanic (9.69% vs. 7.11%; p<0.0517) and less likely to be Black (3.51% vs. 6.85%) (Table 1). 

**Table 1:** Baseline characteristics of CABG patients in the National Inpatient Sample (2008-2020) stratified by history of liver transplant. Significance was determined by a weighted chi-squared test or weighted t-test for categorical and continuous parameters, respectively. CABG: coronary artery bypass grafting

	History of liver transplant	No liver transplant	Absolute difference	P-value
N (weighted)	1,833	2,405,516		
Age (mean in years)	65.16	66.16	1.0	<0.0001
Gender (%)				
Male	81.61 (1,496)	73.66 (1,771,903)	7.95	0.0004
Female	18.39 (338)	26.33 (633,372)	7.94	0.0004
Race (%)				
White	83.02 (1,522)	79.44 (1,910,942)	3.58	0.0882
Black	3.51 (64)	6.85 (164,778)	3.34	0.0109
Hispanic	9.69 (178)	7.11 (171,032)	2.58	0.0517
Other	3.78 (69)	6.60 (158,764)	2.82	0.0298
Comorbidities (%)				
Coronary artery disease	55.96 (1,026)	58.50 (1,407,227)	2.54	0.2974
Hyperlipidemia	56.72 (1,040)	74.26 (1,786,336)	17.54	<0.0001
Hypertension	25.95 (476)	58.45 (2,405,517)	32.50	<0.0001
Diabetes mellitus type 2	57.02 (1,045)	43.39 (1,043,753)	13.63	<0.0001
Congestive heart failure	17.59 (322)	19.67 (473,165)	2.08	0.3442
Anemia	3.26 (60)	3.056 (73,513)	0.20	0.8157
End-stage renal disease	11.21 (205)	2.95 (70,963)	8.26	<0.0001
Obesity	19.00 (348)	23.42 (563,372)	4.42	0.0465
Smoking	12.06 (221)	18.66 (2,405,516)	6.60	0.0021
Gastroesophageal reflux disease	28.39 (520)	21.26 (511,413)	7.13	0.0010
Alcohol use disorder	9.04 (166)	13.44 (323,301)	4.40	0.0174
Hepatic cirrhosis	7.01 (128)	0.49 (11,787)	6.52	<0.0001
Charlson Comorbidity Index (mean)	5.89±0.11	4.16± 0.01	1.73	<0.0001
Insurance (%)				
Medicare/Medicaid	66.24 (1,214)	62.56 (1,504,891)	3.68	0.1488
Self-pay	0.55 (10)	3.40 (81,788)	2.85	0.0027

Patients with a history of LT were more complex (CCI 5.89 vs. 4.16; p<0.001) than patients without a history of LT. Patients with a history of LT also had higher rates of diabetes mellitus type 2 (57.02% vs. 43.39%; p<0.001), end-stage renal disease (11.21% vs. 2.95%; p<0.001), gastroesophageal reflux disease (GERD) (28.39% vs. 21.26%; p<0.001), and hepatic cirrhosis (7.01% vs. 0.49%; p<0.0001). CABG patients with a history of LT were less likely, however, to have hyperlipidemia (56.72% vs. 74.26%; p<0.001), hypertension (25.95% vs. 58.45%; p<0.001), obesity (19% vs. 23.42%; p=0.046), history of smoking (12.06% vs. 18.66%; p<0.01), or alcohol use disorder (9.04% vs. 13.44%; p=0.017). Patients with a history of LT were more likely to have Medicare/Medicaid (66.24% vs. 62.56%; p<0.1488) and less likely to have self-pay (0.55% vs. 3.40%; p<0.0027) (Table 1). 

We found that patients admitted for CABG with a history of LT had significantly higher adjusted odds of mortality (OR 1.76; 95% CI 1.12-2.76; p<0.015), acute kidney injury (AKI) (OR 2.70; CI 2.15-3.38; p<0.0001), and composite outcome (OR 2.08; 95% CI 1.66-2.56; p<0.001). There was no difference in shock (OR 1.10; 95% CI 0.79-1.53; p<0.56) and acute myocardial infarction (OR 0.75; 95% CI 0.50-1.13; p<0.17). They also experienced a longer length of stay (1.67 days; 95% CI 0.21-3.12; p<0.024) and greater hospital charges ($25,975; 95% CI 1912-50037; p=0.029) (Table 2).

**Table 2:** Outcomes for CABG patients with and without a history of liver transplant. Odds ratios were calculated using weighted logistic regression for binary outcomes, while regression coefficients were calculated using weighted linear regression for continuous outcomes. CABG: coronary artery bypass grafting

Outcome	Liver transplant (N=1,833)	No liver transplant (N=2,405,516)	Absolute risk difference	Odds ratio/coefficient	95% CI	P-value
All-cause in-hospital mortality	5.62	2.50	3.12	1.76	1.12-2.76	0.015
Shock	11.16	7.50	3.66	1.10	0.79-1.53	0.56
Acute myocardial infarction	6.95	5.49	1.46	0.75	0.50-1.13	0.17
Acute kidney injury	48.64	18.23	30.41	2.70	2.15-3.38	<0.0001
Composite outcome	55.03	26.48	28.55	2.08	1.66-2.56	<0.0001
Length of stay (mean, days)	12.16	9.60	2.56	1.67	0.21-3.12	0.024
Hospital charges (mean, dollars)	230,309	187,516	42	25,975	1,912-50,037	0.0344

## Discussion

CAD

In this study, NIS data was used to create a large sample size with 1,833 CABG patients with LT and 2,407,349 CABG patients without LT to elucidate the relationship between outcomes of patients receiving CABG with a history of LT. It was found that LT patients had higher adjusted odds of mortality and overall worse outcomes (Table 2). Overall, CAD is the beginning of acute coronary syndrome (ACS), which can require CABG. It was previously thought that ESLD was protective against CAD. However, a common comorbidity of LT patients is CAD, with rates higher than the general population [16-18]. CAD presents in 40-50% of patients with cirrhosis and is associated with systolic and diastolic dysfunction and electrophysiological abnormalities that predispose patients to arrhythmias. This study's percentage was higher, with 55.96% of CABG-LT patients and 58.50% of CABG patients with CAD, which makes sense since these patients had a CABG (Table 1). Cirrhotic cardiomyopathy is believed to resolve 6-12 months post-transplant. However, the heart still has a limited ability to adapt to stress in the peri-transplant period, which can lead to hepatic nephropathy. This is seen with a higher risk of AKI, with a rate of 48.64 in LT patients and 18.23 in non-LT patients (Table 2). Then, 11.21% of LT and 2.95% of non-LT patients had ESRD (Table 1). LT resolves these problems [19,20]. 

Mortality and pre-surgical testing

LT patients with CABG had a twofold higher mortality rate than non-LT patients receiving CABG (Table 2). An increased risk of death after LT was associated with a history of CAD, angiogram confirmation of coronary stenosis, and left ventricular ejection fraction (LVEF) <50% [16,21]. The current recommendation is that patients should undergo ECG and echocardiogram. If the echocardiogram shows LVEF >50%, the patient undergoes stress testing. If the LVEF is <40% with or without symptoms of angina, then the patient undergoes coronary angiography. If the cardiac stress testing is normal, then the patient is cleared cardiologically for an LT. If stress testing shows any wall motion abnormalities, cardiac computed tomography angiography (CTA) is performed [22]. If the cardiac CTA shows sufficient narrowing, the patient undergoes coronary angiography [23,24]. However, patients with ESLD have cirrhotic cardiomyopathy, which consists of high-output heart failure, blunted response to inotropes, and low SVR due to a constant state of vasodilation; these pathophysiologic abnormalities make pharmacological stress tests and echocardiograms inaccurate. 

Physiology of pre-surgical testing

Unless the patient has angina, can walk, or has significant CAD, then the current system of pharmacologic stress testing will likely let patients on the borderline go unnoticed [25]. Even these caveats have limitations; symptoms of angina are different in cirrhotic patients due to symptoms of dyspnea frequently being attributed to volume overload, making it a complex metric to use in this population. Also, in this study, patients with a history of LT had higher rates of diabetes mellitus type 2, end-stage renal disease, and GERD, which are patient populations that frequently have atypical angina (Table 1). The majority of cirrhotic patients are also usually deconditioned and have severe ascites, so their ability to reach the target heart rate in a stress test is difficult, making pharmacologic stress testing the only option, which patients with cirrhosis have a blunted response to inotropes. Finally, the flow reserve is difficult to assess in these patients due to their vasodilated state. Hence, the current use of non-invasive CAD risk assessment is inaccurate in these patients. They are raising the question of whether all of these patients should undergo invasive testing like cardiac catheterization. However, cardiac catheterizations tend to be futile in young patients without symptoms. In this study, patients with a history of LT were more likely to be younger, male, and more complex than patients without a history of LT (Table 1), and CABG patients with a history of LT were less likely to have hyperlipidemia, hypertension, obesity, a history of smoking, or alcohol use disorder, which lends to a lower risk of CAD, which correlates with post-LT patients used in this study. Yet, they are at a higher risk of worse outcomes than their non-LT counterparts when receiving CABG, as shown in this study (Table 2).

CABG and LT timing

Finally, if a cardiac catheterization is performed and a stent is placed, these patients are at a high risk of bleeding, making stent thrombosis more likely. Hence, should certain CAD be prohibitive to LT? When should percutaneous coronary intervention (PCI) or CABG be performed? Compared to the current study, the research regarding CABG and LT is on when to perform the CABG. Should it be done in a patient who is a candidate for transplant or in the process of receiving a liver or has received an LT? Many articles have been published with small patient populations exploring this matter. 

Many cases have been shown to support CABG before LT poorly. A retrospective analysis at Indiana University School of Medicine pulled records for pre-transplant and non-transplant patients undergoing CABG. They found that 30-day mortality and neurological events were higher in pre-transplant patients undergoing CABG and most patients did not go for organ transplants post-CABG [26]. The CREDO-Kyoto registry of 9877 patients found coronary revascularization in patients with liver cirrhosis did not show better survival outcomes [27]. In another article, two cases of CABG before LT were performed; they waited at least 10 days before performing LT and had favorable results [28]. The reason for the difficulty in performing CABG before LT is likely due to the complexity of ESLD patients, leading to prolonged or minimal recovery.

Multiple case reports and studies were shown with better support for CABG and LT performed together. A few case reports of LT with CABG performed simultaneously were successful [29,30]. In a retrospective study of 400 patients, five had CABG and LT simultaneously; at 25 months follow-up, it had 80% patient survival [31]. Thirty-one patients at another institution who had LT+CS had 74.2% and 55% one- and five-year survival rates, respectively. Factors like old age, non-alcoholic steatohepatitis (NASH)-cirrhosis, CABG-based CS, and preoperative renal dysfunction were found to affect rates of survival [32]. A potential complication of performing LT and CABG simultaneously is that opening the chest can become complicated by creating stress on the sternal break, which creates a wound that is difficult to close, risking the development of osteomyelitis [33]. Also, the changes in hemodynamics, the average time of surgery reaching 12+ hours, and changes in fluid mechanics with CABG and LT can lead to a prolonged recovery. 

Finally, a few studies showed that CABG post-LT had some beneficial results. A study found that 12 patients from 1998 to 2004 underwent surgical therapy for CVD with a history of LT. These patients had transient allograft dysfunction and 91% and 67% one- and five-year survivals, respectively [34]. Another study of 12 patients with LT after CABG had promising results [35]. In another study, they looked at 11,190 patients; 129 had solid organ transplants and underwent CABG, and 27 had LT. They were found to have favorable five-year mortality outcomes but had more readmissions than non-transplant patients [36]. In this study, the NIS data was organized for admissions with a history of LT in patients admitted for CABG. The data can comment on the outcomes of CABG post-LT. Still, information on the exact timing cannot be elucidated because the current data in the NIS database do not document the procedure timing. 

Length of stay and hospital charges

LT patients had longer lengths of stay and, in turn, greater hospital charges (Table 2), likely due to being more complex, seen with elevated CCIs (Table 1 and Table 2). Another proposed reason for a greater length of stay in LT-CABG is that some cardiopulmonary bypass patients experience hepatic injury due to systemic inflammatory response and oxidative stress from decreased hepatic perfusion [37,38]. This can result in centrilobular sinusoid ischemia and reperfusion injuries post-CABG [39]. Liver graft regeneration is generally a fickle matter; it requires adequate portal flow and portal vein pressure. A case report of a live donor liver transplant (LDLT) found that time lag ligation (TLL) with a coronary artery bypass occluder increased portal flow without a need for invasive laparotomy [40]. The readily available surgical device is a worthwhile alteration that should be looked into. No information exists to compare various outcomes, as only a case report exists.

Limitations

The limitations of this study are based on the NIS database's inherent capabilities. With this database, the timing cannot be elucidated on when the LT and CABG were performed, and there is an inability to trace specific patient outcomes longitudinally since it only covers the patient during the hospital stay. Other databases that detail transplants, like the Organ Procurement and Transplantation Network (OPTN) and NIS readmission databases, could be used to explore this subject further. Then, this database's data points are based on admissions rather than on a specific patient since the data is removed from all patient-identifying factors. Hence, there is a possibility of over-calculation of the disease burden due to multiple inpatient admissions of the same patient. On admission, one cannot differentiate the main reason for admission; one can only differentiate between LT and CABG based on ICD codes. The reason for an LT cannot be delineated with this dataset nor the time to an LT. Admission data was identified in the NIS using ICD-9 and ICD-10 diagnosis codes; subtle differences exist between the nature of coding using ICD-9 and ICD-10, which can alter the data when combined to analyze trends. Also, the specific causes of death cannot be elucidated. Furthermore, the NIS does not detail important clinical predictors, such as the duration of comorbidities among the observed patient's intraoperative complications. The NIS does not provide detailed information about specific management, including treatment medications and lab values. 

## Conclusions

The study expanded upon the world of LT research with a large sample size, creating a powerful study using the NIS database. In this study, we were able to elucidate various previously undetailed outcomes due to the rarity and complexity of these patients. We found that CABG patients with a history of LT had roughly twofold higher odds of mortality, roughly threefold higher odds of AKI, and twofold higher odds of composite outcomes than CABG patients without LT. This corresponded to longer lengths of stay and increased hospital charges. Overall, the performance of CABG on ESLD favors it being done during or after LT, but these patients are still at a high risk of mortality. The decision requires the communication and coordination of a multidisciplinary team. Future research, such as prospective cohort studies, should be conducted to determine the optimal timing of CABG relative to LT and evaluate specific intraoperative strategies or postoperative monitoring protocols for CABG in LT patients. Also, better efforts must be made to avoid the scenario through more accurate pre-surgical workup before an LT. More research needs to be performed to elucidate better when cardiac catheterization should be performed. 
